# Canine fossa puncture in endoscopic sinus surgery: report of two cases^☆^

**DOI:** 10.1016/j.bjorl.2017.03.001

**Published:** 2017-03-22

**Authors:** Federico Sireci, Matteo Nicolotti, Paolo Battaglia, Raffaele Sorrentino, Paolo Castelnuovo, Frank Rikki Canevari

**Affiliations:** a University of Palermo, Department of Experimental Biomedicine and Clinical Neurosciences (BioNeC), Otorhinolaryngology Section, Palermo, Italy; b SS Antonio Biagio e Cesare Arrigo Hospital, Otorinolaryngology Section, Alessandria, Italy; c University of Insubria, Department of Otorhinolaryngology, Varese, Italy

**Keywords:** Canine fossa puncture, Middle meatal antrostomy, Maxillary sinusitis, Angled microdebrider, Punção da fossa canina, Antrostomia meatal média, Sinusite maxilar, Microdebridador angular

## Abstract

**Introduction:**

Chronic rhinosinusitis with nasal polyposis is a common chronic disease that often affects maxillary sinus. Endoscopic sinus surgery is the most common procedure for treating the majority of maxillary sinus lesions.

**Objective:**

To demonstrate the role of canine fossa puncture during endoscopic sinus surgery procedure in patients with severe maxillary sinus disease.

**Methods:**

We present 2 cases where canine fossa puncture has been performed as method to obtain a complete access to the maxillary antrum.

**Results:**

According our experience, 2 cases on 296 endoscopic sinus surgery (0.6%) where antrostomy and used of angled microdebrider were not sufficient, canine fossa puncture has been performed as an alternative method to obtain a complete access to the maxillary antrum.

**Conclusion:**

Although the advent of endoscopic sinus surgery, our cases support the fact that actually canine fossa puncture is a minimally invasive technique useful in selected cases.

## Resumo

**Introdução:**

A rinossinusite crônica com polipose nasal é uma doença crônica comum que frequentemente afeta o seio maxilar. A cirurgia endoscópica sinusal é o procedimento mais comum para tratar a maioria das lesões do seio maxilar.

**Objetivo:**

Demonstrar o papel da punção da fossa canina durante o procedimento de cirurgia endoscópica sinusal em pacientes com doença grave do seio maxilar.

**Método:**

Apresentamos 2 casos em que a punção da fossa canina foi realizada como método para obter acesso completo ao antro maxilar.

**Resultados:**

De acordo com nossa experiência, 2 casos em 296 cirurgia endoscópica sinusal (0,6%) onde antrostomia e o uso de microdebridador angular não foram suficientes, a punção da fossa canina foi realizada como um método alternativo para obter acesso completo ao antro maxilar.

**Conclusão:**

Apesar do advento da cirurgia endoscópica sinusal, os nossos casos apoiam o fato de que a punção da fossa canina é uma técnica minimamente invasiva útil em casos selecionados.

## Introduction

Chronic rhinosinusitis (CRS) with nasal polyposis (NP) is a common chronic disease that seriously affects the quality of life.^1^ Obliteration of the ostiomeatal unit is the most common factor influencing the pathogenesis of this inflammatory process.^2^ Endoscopic sinus surgery (ESS) is the most common procedure for treating CRS that is refractory to medical treatment.

Although the majority of maxillary sinus lesions can be removed through the widened natural ostium performing partial uncinectomy and middle meatal antrostomy (MMA), some patients have extensive disease that is difficult to handle purely endoscopically.

In fact endoscopic access by natural ostium only allow to clear the posterior lateral wall, the posterior region of the roof, and the posterior wall of the maxillary sinus but no anterior and inferior regions. Failure to remove massive polyposis and fungal debris from the maxillary sinus may result in an early postoperative recurrence of symptoms and disease for the patient.^3^

One solution is the traditional Caldwell Luc approach performed through the anterior wall of the maxillary sinus. This is, however, associated with significant morbidity such as facial numbness or paresthesia (9%) for damage of the infraorbital and anterior superior alveolar nerves, oroantral fistulas (1%), gingivolabial wound dehiscences (1.5%), and dacryocystitis (2.5%). This technique has been superseded by endoscopic medial maxillectomy useful to treat benign tumors, not inflammatory disease.^4^ Canine fossa puncture (CFP) has been proposed as an alternative method of obtaining access to the maxillary antrum.

Although a few studies have demonstrated the benefits of CFP in management of the severely diseased maxillary sinus, the efficacy and superiority of this method compared with conventional MMA require further investigation.^5^

The aim of this study is to review the literature about the indications of CFP in patients with severe maxillary sinus disease and compare this surgical procedure with maxillary sinus clearance through MMA.

## Presentation of cases series

We reviewed all endoscopic sinus surgery (ESS) performed at our department in 2015. A total of 296 endoscopic sinus surgeries were performed during this period. Of these, combined approach endoscopic sinus surgery and canine fossa puncture (CFP) was performed in 2 (0.6%) cases when there was difficulty in visualization and clearance of disease.

In particular, CFP consist in a trocar placed in the canine fossa. The landmarks in canine approach are: the mid pupillary line and horizontal line running along the lower border of nasal ala and lateral aspect of canine fossa high above the 3rd and 4th teeth (canine and premolar), infero lateral to infra orbital foramen. The trocar should be aimed toward the maxillo ethmoid angle to avoid pterygopalatine fossa and orbital lesions. In most patients the trocar was inserted using a gentle twisting motion. In some patients with thicker bone, gentle tapping with a hammer was required for the trocar to be inserted.^6^, ^7^ After removal of the trocar a 4 mm microdebrider blade was placed through the passage created by the trocar. The microdebrider blade was visualized in the maxillary sinus with a 40° or 70° endoscope via the middle meatal antrostomy. Polyps and diseased tissue can be removed from the maxillary sinus using the microdebrider. Both cases treated by CFP resulted free of complications after surgery and free of disease during follow-up.

### Case 1

**Figure 1:**
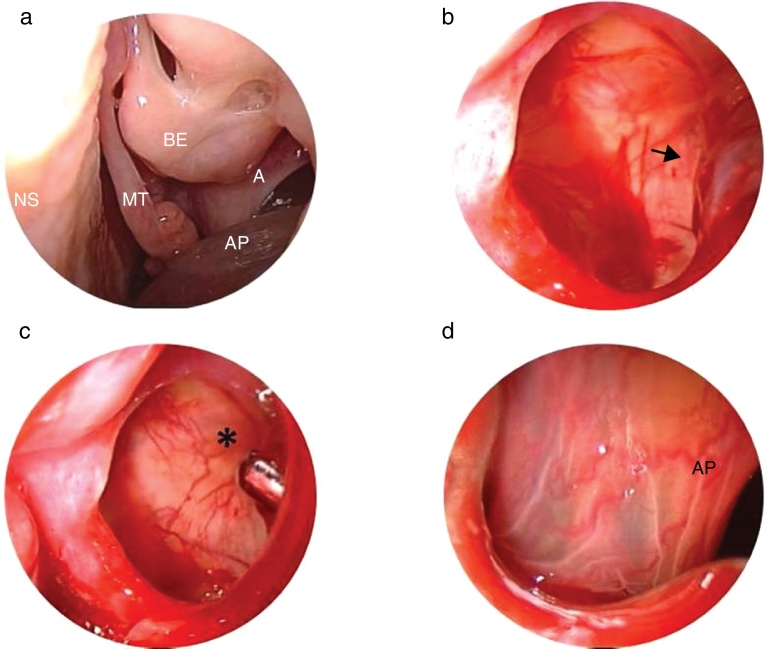
(a) Antrochoanal polyp emerging from the middle antrostomy of the left maxillary sinus is noted on the endoscopic examination. (b) The base of the polyp (black arrow) on the anterior wall of the maxillary sinus viewed using 45° endoscope. (c) Microbedrider (asterisk) introduced inside the maxillary sinus by the trocar viewed using 45° endoscope. (d) Polyp viewed by trocar using 0° endoscope. AP, antrochoanal polyp; A, antrostomy; MT, middle turbinate; NS, nasal septum; BE, bulla ethmoidalis.

A 20-year-old Caucasian female was admitted to our Department on July 2015. She was nonsmoker and denied alcohol consumption. She reported paracetamol and dried fruit allergy. Three years ago, underwent to functional endoscopic sinus surgery (FESS) for presence of left antrochoanal polyp. Since 3 months, she complained a left nasal obstruction without other symptoms. Therefore, she has been subjected to nasal endoscopy that evidenced a presence of left antrochoanal polyp (Fig. 1) confirmed at cranio-facial computerized tomography (CT). In August 2015 a revision FESS was performed. Due to the fact that polyp implant was in anterior region of maxillary sinus, a canine fossa puncture was performed, despite the use of curved blades. The nasal pack was removed the day after and the patient discharged. Seven days of antibiotics therapy amoxicillin/clavulanate combination and salin nasal irrigation was recommended. The patient underwent new examination at 6 months remaining free of disease.

### Case 2

**Figure 2:**
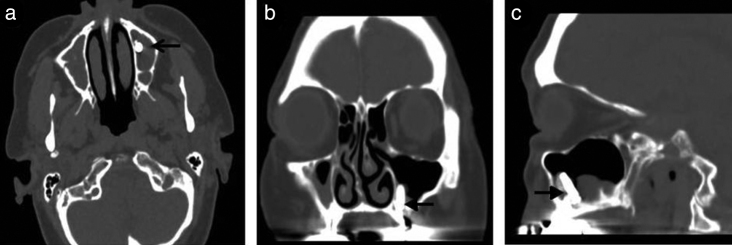
Computed tomography (CT) scan showed dental implant (arrow) inside left maxillary sinus in axial (a), coronal (b) and sagittal (d) view.

**Figure 3:**
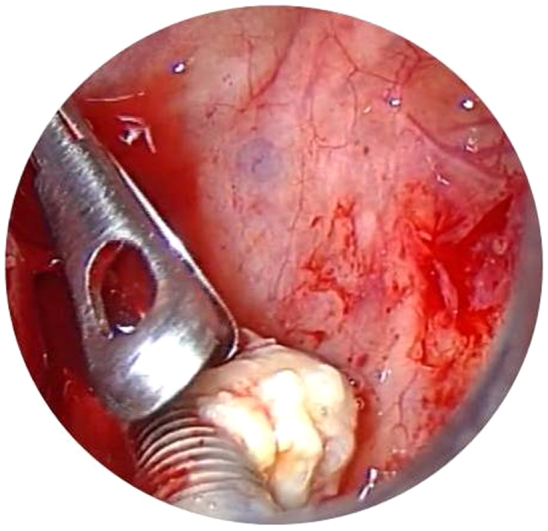
Dental implant view by trocar using 0° endoscope.

A 77-year-old Caucasian woman was admitted to our Department because of left purulent rhinorrhea and paresthesia in left maxilla region since 3 months. She was subjected to dental implant on left maxilla arch in March 2015. Her past medical history was remarkable; she had a positive remote history for chronic lymphoblastic leukemia B cell resolved, splenectomy performed for spontaneous splenic rupture, hysterectomy and atrial fibrillation. An anterior rhinoscopy displayed the presence of pus filling his whole nasal cavity, which was originating from the ostio-meatal complex. During oral evaluation an oro-antral fistula was detected at the site of previous dental extraction. CT scan without contrast enhancement was performed and showed complete opacification of the left maxillary sinus and an interruption of sinus floor corresponding to alveolar process with dental implant inside sinus (Figure 2, Figure 3).

In April 2015 patient was subjected to MMA in order to remove dental implants but the procedure could not be completed transnasally neither with the use of curved blade so a CFP was required in order to remove the foreign body. Nasal packing was removed 1 day postoperatively, and the patient was discharged. After surgery, we prescribed saline irrigation and paracetamol. Patient has been visited in our section at 10 days, then at 4 weeks and finally at 6 months. Meticulous endoscopic dressing and saline irrigation were performed until the cavity was healed. To date patient is free of disease.

## Discussion

Nowadays middle meatal antrostomy (MMA) is considered the gold standard I the treatment of maxillary sinusitis. Anyway critical areas such as inferior, lateral, anterior wall, recessus zygomaticus and recessus alveolaris and recessus prelacrimalis of maxillary sinus are difficult to approach. For this reason medial maxillectomy or external approaches such as Caldwell Luc approach or CFP are required.^6^

The efficacy of this maneuver on the outcome after FESS has been the subject of a small number of studies. Lee et al. have compared the results of performing a canine fossa puncture with clearance of polyps via a middle meatal antrostomy. No benefit of the canine fossa procedure over conventional middle meatal antrostomy was seen after 12 months follow up, however series are small in both groups (11 CFP vs. 13 MMA). The authors concluded that although canine fossa puncture is a useful method for removing severe mucosal disease that cannot be reached through the MMA, it does not guarantee a better subjective or objective surgical outcome in patients with nasal polyposis.^7^ However, Seiberling et al. in a case control studies have found that patients with the same disease who had a canine fossa puncture had a better outcome than those who did not. In fact, all patients were administered the chronic sinusitis survey (CSS) that evidenced a subscore better in the CFP group (Mann–Whitney test, *p* = 0.02).^8^

Many authors studied the maxillary sinus area explored by endoscopy.

Beswick et al. quantified the maxillary sinus volume and mucosal surface area (SA) that is accessible endoscopically via a middle meatal antrostomy in eight cadaver maxillary sinuses configured with image guidance software. In particular the authors demonstrated that mean maxillary sinus volume was 16.5 ± 2.5 cm^3^ and mean SA was 31.0 ± 2.3 cm^2^. The 15°, 40°, 70° and 120° microdebriders accessed an average of 10%, 25%, 41%, and 66%, respectively, of the SA, and of 2%, 9%, 17%, and 36%, respectively, of the volume. There was a trend toward improved accessibility of the superior half versus the inferior half of the maxillary sinus. When instruments of different degrees were combined to maximize accessibility, 81% of the SA of the sinus could be accessed. However authors did not quantify volume and area of maxillary sinus exposed.^9^

Feldt et al. in a sample of cadavers showed that a significant greater amount of debris was left after the transnasal endoscopic technique (TN) approach compared with canine fossa trephination (CFT) (3.88 cm^3^ vs. 2.88 cm^3^, *p* = 0.015). Median blade utilization was significantly higher with the TN approach vs. CFT (4 vs. 1, *p* < 0.002).

Our experience demonstrated as, although using new curved blades, critical areas such as antero-medial portion of MS cannot be reached and treated by antrostomy and therefore CFP has to be performed. According to literature, we performed CFP only in unilateral lesions. In fact Byun et al. assigned randomly twelve patients to the CFP and thirteen to MMA groups who completed the follow-up, questionnaires (SNOT-20 and VAS), and postoperative CT evaluation. SNOT-20 and VAS scores improved significantly at 3, 6, and 12 months postprocedure in both groups. However, significant improvement of SNOT-20 at 12 months and VAS scores for purulent discharge, foul odor, and postnasal drip at 6 and 12 months were observed in the CFP group compared with the MMA group. Postoperative CT scan evidenced that the volume of mucosal thickening was significantly greater in the MMA group than in the CFP group. In addition, CFP was not effective in patients with bilateral CRS and diffuse NP but provided better management and subjective and objective surgical outcomes in patients with unilateral CRS accompanying NP. Among unilateral NP, there is anthrocoanal polyps (ACP).

ACP is relatively unusual in the sinonasal tract. It occurs predominantly in children and young adults and originates from the maxillary antrum with extension into the nasopharynx or oropharynx. Simple excision of the polyp via limited surgical procedures such as simple polypectomy results in high recurrence rates. Complete removal of the polyp in the antrum, including its site of origin, is essential to minimize recurrence. However, a broad base such as a lateral, inferior, or anterior location of the polyp makes eradication difficult via a MMA. In this situation, some authors choose the traditional Caldwell-Luc operation, even in children.^10^ However, the increased morbidity of this procedure, including facial numbness, facial swelling, risk for injury of the tooth buds and roots, and possible asymmetric facial growth, makes it less favorable than endoscopic sinus surgery. In fact, we preferred CFP as alternative method of accessing almost the entire maxillary antrum in young/adult population. Few studies have reported the efficacy of the CFP approach in treating ACP in the pediatric population (≤15 years old) and the long-term effect on changes in the maxillary sinus volume and surgical outcome has not been determined. Jae Yong Lee et al. studied a little sample of seven patients with ACP removed via the CFP approach. After a mean follow-up period of 43.9 months, the authors compared the changes in the maxillary sinus volume between the operated and normal sides, using the pre- and postoperative CT data. No contractures or decrease in the maxillary sinus volume on the diseased side were observed in any of the patients on postoperative CT. None of the patients showed evidence of recurrence on the endoscopic and CT examinations and only two patients complained of mild facial swelling and tingling sensation, which resolved spontaneously within 2 weeks.^11^ Therefore CFP can be performed to treat ACP in young but also in children when MMA is not sufficient.

Important indications to CFP are chronic odontogenic sinusitis (COS). Approximately 5–15% of the population suffers from chronic rhinosinusitis, and in 10–12% of them, it is of dental origin.^12^ It is produced by periapical granulomas or small inflammatory cysts of the molars or bicuspids, chronic oroantral fistula (OAF), large odontogenic cysts occupying a great part of the maxillary sinus, and foreign bodies (dental fillings, teeth roots, and implants) pressed throughout the root canal or fistula into the antrum. We showed a case of implant migrate in left maxillary sinus not accessible from antrostomy by curved instruments because placed in medial wall of maxillary sinus.

Venetis et al. presented 20 cases with odontogenic sinusitis, 5 (25%) in which a foreign body was removed from maxillary sinus by combined transnasal and canine fossa endoscopic approach. The surgical procedure was performed with conventional instrumentation through the widened antrorhinostomy or through a bony window at the anterior sinus wall. At the end of the procedure, the window was repositioned and stabilized with two to three absorbable sutures. In two cases, with sizeable foreign bodies, the preservation of the bony window was impossible, and a modified Caldwell–Luc operation was performed with preservation of the healthy sinus mucosa.^13^ According to Barziliai et al. we avoid Caldwell–Luc operation because it is associated with significant morbidity such as facial numbness or paresthesia (9%) for damage of the infraorbital and anterior superior alveolar nerves, oroantral fistulas (1%), gingivolabial wound dehiscences (1.5%), and dacryocystitis (2.5%).^14^ Actually Caldwell-Luc procedure is rational in cases of fungal disease and in endoscopic medial maxillectomy for treating inverted papilloma. In fact in our case, a wide MMA combined with CFP was enough to remove dental implant.

In literature, different opinions about approach to fungal maxillar sinusitis are reported. We did not show any case of CFP in fungal disease because in most of the patients it can be removed through the MMA using various curved instruments and saline irrigation without great difficulty. Also, if fungal invasion into sinus tissue is confirmed by histological examination, a systemic antifungal treatment postoperatively is paramount to reduce disease and potential recurrences.^15^

Instead there are no indications to perform CFP and/or Caldwell-Luc approach in maxillar sinus malignant tumor. According to literature, endoscopic medial maxillectomy can be considered as first choice in difficult maxillary sinus tumor removal because an unnatural opening like CFA or Caldwell Luc in locally invasive disease with malignant potential as the tumor can extend through this opening in recurrent cases.^16^

## Conclusions

Although the advent of ESS, our cases support the fact that actually canine fossa puncture is a minimally invasive technique useful in selected cases. In fact, only in 0.6% of cases we used this technique without postoperative complications.

## Conflicts of interest

The authors declare no conflicts of interest.
